# Neutralizing IL-15 Inhibits Tissue-Damaging Immune Response in Ex Vivo Cultured Untreated Celiac Intestinal Mucosa

**DOI:** 10.3390/cells14030234

**Published:** 2025-02-06

**Authors:** Vera Rotondi Aufiero, Giuseppe Iacomino, Giovanni De Chiara, Errico Picariello, Gaetano Iaquinto, Riccardo Troncone, Giuseppe Mazzarella

**Affiliations:** 1 Institute of Food Science, National Research Council (ISA-CNR), 83100 Avellino, Italy; vera.rotondiaufiero@isa.cnr.it (V.R.A.);; 2 European Laboratory for the Investigation of Food Induced Diseases (ELFID), Via Pansini 5, 80131 Napoli, Italy; 3 Anatomic Pathology Unit, San G. Moscati Hospital, 83100 Avellino, Italy; 4 Gastroenterology Unit, S. Rita Hospital, 83100 Avellino, Italy; 5 Department of Medical Translational Sciences, University Federico II, 80131 Naples, Italy

**Keywords:** IL-15, celiac disease, cytotoxicity, therapy

## Abstract

In celiac disease (CeD), interleukin 15 (IL-15) affects the epithelial barrier by acting on intraepithelial lymphocytes, promoting interferon γ (IFN-γ) production and inducing strong cytotoxic activity as well as eliciting apoptotic death of enterocytes by the Fas/Fas ligand system. This study investigates the effects of a monoclonal antibody neutralizing the effects of IL-15 (aIL-15) on tissue-damaging immune response in untreated CeD patients by using an organ culture system. Jejunal biopsies from 10 untreated CeD patients were cultured ex vivo with or without aIL-15. Epithelial expressions of CD95/Fas, HLA-E and perforin were analyzed by immunohistochemistry. Apoptosis was detected in the epithelium by using the terminal deoxynucleotidyl transferase-mediated dUTP nick-end labeling (TUNEL) assay. Additionally, the surface epithelium compartment of ex vivo cultured biopsy samples was isolated by laser capture microdissection (LCM). RNA from each LCM sample was extracted and the relative expression of IFN-γ was evaluated by quantitative reverse transcriptase-PCR (qRT-PCR). Biopsies cultured with the aIL-15 antibody showed a reduction in Fas, HLA-E and perforin epithelial expression, as well as a decrease in epithelial TUNEL+ cells compared to biopsies cultured without the aIL-15 antibody. Moreover, downregulation of epithelial IFN-γ expression was recorded in biopsies incubated with aIL-15, compared to those cultured without aIL-15. Our findings suggest that neutralizing the effects of IL-15 in ex vivo cultured untreated CeD intestinal mucosa could block apoptosis by downregulating Fas and HLA-E expression and the release of cytotoxic proteins, such as perforin. Furthermore, it can dampen the hyperactive immune response by reducing IFN-γ expression. More generally, our study provides new evidence for the effects of anti-IL-15 neutralizing monoclonal antibodies in preventing or repairing epithelial damage and further supports the concept that IL-15 is a meaningful therapeutic target in CeD, or inflammatory diseases associated with the upregulation of IL-15.

## 1. Introduction

Celiac disease (CeD) is defined as a chronic small-intestinal, immune-mediated enteropathy precipitated by exposure to dietary gluten in genetically predisposed individuals [1]. The immune response against gliadin peptides is mediated through cytokines produced via both innate and adaptive immune branches [2]. In CeD, epithelial cells are likely destroyed via toxic gliadin peptides, such as 19-mer, that might activate the innate immune system, upregulating interleukin (IL)-15 secretion [3]. Immuno-adaptive peptides, such as the 33-mer, can enter the lamina propria, where the HLA class Ⅱ DQ2+ or DQ8+ molecules present these peptides to T cells, which activate gluten-reactive Th1 and Th17 helper T cells producing high levels of proinflammatory cytokines such as IFN-γ [4].

A gluten-free diet (GFD) is the only available treatment for CeD, but many patients do not respond completely clinically or histologically. Furthermore, following a strict GFD still now can be psychologically and socially challenging because of its restrictive nature. Hence, efforts are ongoing to develop therapeutic strategies beyond GFD. The most interesting treatment strategies include induction of tolerance, enzymatic degradation of gluten by glutenases, restoration of the epithelial tight junction barrier function, inhibition of tissue transglutaminase and the use of monoclonal antibodies targeting human leukocyte antigen-mediated gliadin peptide presentation and cytokines involved in the pathogenesis of CeD [5]. Among cytokines, IL-15 is a molecule that plays a crucial role in the pathogenesis of CeD.

IL-15 interacts with other cytokines to promote the maturation of dendritic cells, the proliferation of T and B cells, the cytotoxicity of NK and CD8+ T cells and the production of proinflammatory cytokines, such as tumor necrosis factor (TNF)-α and IL-1β [6]. Such cytokines are involved in maintaining immune homeostasis, and their altered expression has been involved in promoting the pathology of proinflammatory or autoimmune-related diseases, including type-1 diabetes (T1D), psoriasis and rheumatoid arthritis, beyond CeD. For all these broader implications of IL-15 signaling in the progression of these diseases, it was shown to be used as an immunotherapeutic target [7].

A series of therapeutic agents that inhibit IL-15 action have been introduced, including the soluble IL-15 receptor (IL-15R) α chain, mutant IL-15 and antibodies directed against both the IL-15 cytokine and IL-15R β subunit [8]. However, beyond IL-15, other cytokines are also involved in the loss of immune homeostasis. Therefore, it is crucial to understand the downstream consequences of IL-15 blocking. We cannot exclude that combination therapies or therapies targeting common signaling pathways may be necessary to achieve a therapeutic effect. In this direction, the use of the organ culture system is predicted to be very valuable.

Of note, in CeD, IL-15 plays a critical role through the activation of intraepithelial cytotoxic T lymphocytes that lead to villous atrophy [9,10,11,12]. As previously shown by many groups, including ours, IL-15 not only enhances the cytotoxicity of CD8+ intraepithelial lymphocytes (IELs) and NK cells in CeD but also impairs the functions of T regulatory (Treg) cells [13,14], suggesting that such cytokines promote the dysregulation of immune mechanisms that contribute to the immunopathogenesis of the disease.

Several studies have analyzed the efficacy of innovative therapeutic strategies for CeD, among which the effect of monoclonal antibodies neutralizing IL-15 protein on duodenal biopsies from CeD patients by an ex vivo approach, uncovering relevant insights into the pathogenesis of the disease [15].

Maiuri et al. [3] have shown that a non-immunodominant peptide of gliadin induced a rapid expression of IL-15 and enterocyte apoptosis and that such activity was affected by IL-15 inhibition. Hue and colleagues [10] revealed that, in treated CeD, gliadin induces MICA expression at the surface of gut epithelial cells via a pathway involving IL-15, providing an epithelial target to cytotoxic CD8+ IELs. Moreover, MICA expression was sensitive to inhibition by IL-15. More recently, it was shown that IFN-γ produced following stimulation with gliadin in the supernatants of duodenal organ cultures generated from biopsies of untreated CeD patients was significantly reduced in the presence of BNZ-2, a peptide designed to inhibit IL-15 and IL-21 [9]. These findings lead to the hypothesis that a therapy aimed at neutralizing the effect of IL-15 may prospectively restore mucosal injury in the intestinal mucosa of CeD patients.

In this study, we investigated, for the first time, the effect of a neutralizing anti-IL-15 monoclonal antibody (aIL-15) in preventing epithelial damage in the intestinal mucosa of untreated CeD patients by analyzing the expression of perforin, CD95/Fas and HLA-E by immunohistochemistry. In addition, cell apoptosis, by terminal deoxynucleotidyl transferase-mediated dUTP nick-end labeling (TUNEL) assay and the hyperactive immune response through analysis of epithelial IFN-γ expression by qRT-PCR, was studied.

## 2. Materials and Methods

### 2.1. Patients

Patients underwent jejunal biopsy for routine checkups, and they gave their full informed consent in this study. This study was approved by the ethics committee of the San G. Moscati Hospital (Avellino, Italy, n°CECN/809) and complies with the provisions of the Declaration of Helsinki.

Ten patients with untreated CeD (n.3 male and n.7 female; median age 39 years; range 18–45 years), diagnosed based on ESsCD criteria [16], were recruited. All untreated CeD patients were positive for serum anti-endomysial antibodies and had no comorbidities. Phenotypic data of untreated CeD patients are reported in Supplementary Table S1.

### 2.2. Organ Culture and Immunohistochemical Analysis

During endoscopy, at least four biopsies were taken from the jejunal mucosa of untreated CD patients. Immediately after the endoscopy, one specimen was embedded in an optimal cutting temperature compound (OCT; Killik, Bio-Optica, Milan, Italy) and stored in liquid nitrogen until use. Two mucosal specimens were cultured as described elsewhere [17] to perform organ culture experiments. Briefly, jejunal biopsies were placed on a stainless-steel mesh positioned over the central well of an organ culture dish (Becton Dickinson, New York, NY, USA) with the mucosal surface oriented on the top of the well. The biopsy specimens were cultured for 24 h at 37 °C with medium alone or with anti-IL-15 monoclonal antibody (Catalog # MAB2471, 15 μg/mL; R&D Systems, Minneapolis, MN, USA). The medium was composed of RPMI 1640 (80%; Sigma, Milan, Italy) supplemented with fetal bovine serum (15%; Life Technologies-GibcoBRL, Milan, Italy), L-glutamine (2 mM; Life Technologies-GibcoBRL), penicillin (100 U/mL) and streptomycin (100 μg/mL) (Life Technologies-GibcoBRL). After the incubation time had passed, the tissues were embedded in OCT, snap-frozen in liquid nitrogen and processed for immunohistochemistry or stored at −80 °C for downstream molecular analysis.

For immunohistochemical analysis, cryostat sections (5 μm) were fixed in acetone and stained with the following monoclonal antibodies (mAbs): anti-CD95/Fas (Catalog #555671, Becton Dickinson; 1:100), anti-HLA-E (Catalog #11-361-C100; Ex-bio Praha; 1:100) and anti-perforin (Catalog #556434, Becton Dickinson, Buccinasco, Milan, Italy; 1:100). Antibody binding was visualized by using the peroxidase EnVision System (Dako, Carpinteria, CA, USA). The sections were finally stained with Mayer’s hematoxylin. The staining of the epithelial cells for CD95/Fas and HLA-E was graded as weak (+) = 1, moderate (++) = 2, or strong (+++) = 3. Isotype control sections were prepared under identical immunohistochemical conditions, as described above, replacing the primary anti-CD95/Fas, anti-HLA-E and anti-perforin antibodies with a purified, normal mouse IgG control antibody (DAKO).

Cell apoptosis was detected on frozen tissue fixed with 4%paraformaldehyde using the TUNEL assay (Roche, Mannheim, Germany). The reagents were incubated for 1 h at 37 °C. Finally, the samples were counterstained with ToPro-3 and imaged with a confocal microscope (Leica SP8, Leica Microsystems Srl, Milan, Italy). All slides were blindly analyzed by two observers. The density of the perforin and apoptotic cells in the intraepithelial compartment was determined by counting the number of stained cytotoxic granules and the number of TUNEL+ cells per mm epithelium. The counts were independently analyzed in a blind manner by two observers.

### 2.3. Laser Capture Microdissection, RNA Extraction and Retrotranscription

Frozen biopsy specimens cultured with and without aIL-15 from five untreated CeD were cut into 10 μm sections using a cryostat (Leica CM1850; Leica Microsystems, Wetzlar, Germany) and collected on RNase-free membrane slides (PEN-membrane, 1 mm glass, Carl Zeiss MicroImaging, Munich, Germany). The sections were immediately fixed in ice-cold EtOH 70% for 2 min and then they were rehydrated in ice-cold diethylpyrocarbonate (DEPC) water. The following steps were dehydration in graded EtOH for 60 s and xylene for 2 min. The slides were then air-dried for 3 min and microdissected by a laser capture microdissector (LCM) to collect an area of 2 mm^2^ from the surface epithelium (SE) compartment. Samples obtained by laser microdissection were processed in the presence of the 4 U RNasin RNase inhibitor (Promega, Italy), and total RNA was extracted by using the PicoPure RNA isolation kit according to the manufacturer’s protocol (Arcturus, Thermo Fisher Scientific, Milan, Italy).

The RNA was eluted in 12 µL DEPC water. The Experion automated electrophoresis station (BioRad) was used to assess RNA quality, and a fluorimetric approach was used on a Qubit^®^ ver.3 instrument (Thermo Fisher Scientific, Milan, Italy) to evaluate RNA. The SuperScript VILO cDNA Synthesis Kit (Thermo Fisher Scientific, Italy), with random hexamer primers according to the manufacturer’s instructions, was used to reverse transcribe isolated RNAs.

### 2.4. qRT-PCR

Expression analysis for IFN-γ cDNAs was performed by SYBR green (PowerSYBR Green PCR Master Mix, Applied Biosystems, Milan, Italy) by using qRT-PCR assays based on an ABI PRISM 7000 SDS instrument (Applied Biosystems). A total of 2 µL of the cDNA dilutions was used in triplicate in a final volume of 35 µL for the PCR reactions. In each quantitative PCR run, a standard curve was generated with serial dilutions of cDNA containing a known quantity of each transcript. The specificity of the amplification products was assessed by a melting curve analysis. Preliminary experiments were performed to establish the optimal dilution of cDNAs to obtain a PCR product within the linear phase of the amplification. The samples where both housekeeping gene and gene had a sigmoid-shaped curve between Ct values from 15 to 36 were useful for this study. Samples that did not meet the RNA quality and quantity requirements were excluded from this study. Primers were synthesized by Sigma-Aldrich. Oligonucleotides were designed to (a) generate amplicons of 50–150 bp from specific National Center for Biotechnology Information (NCBI) reference sequences to (b) span exons and were blasted through NCBI GenBank to ensure lack of homology to other known human cDNA sequences. Gene expression was normalized to the level of the housekeeping gene glyceraldehyde-3-phosphate dehydrogenase (GAPDH).

Primer sequences are reported in Table 1.

### 2.5. Data and Statistical Analysis

Immunohistochemical data are presented as mean values ± SD; a paired two-tailed Student’s *t*-test was used to calculate *p*-values within the same individuals, and an unpaired two-tailed Student’s *t*-test was used to calculate *p*-values between study groups. *p*-values < 0.05 were considered statistically significant.

The relative expression of transcripts was calculated by the ΔΔCt method using the Data Assist Software v3.01 (Applied Biosystems). Statistical analyses were performed with GraphPad Prism 6 (GraphPad Software, CA, USA). Differences between groups were compared using paired two-tailed Student’s *t*-tests. Statistical significance was achieved when *p* < 0.05.

## 3. Results

### 3.1. Blocking IL-15 Downregulates the Epithelial Expression of Apoptotic Molecules in Untreated CeD Mucosa

**Figure 1:**
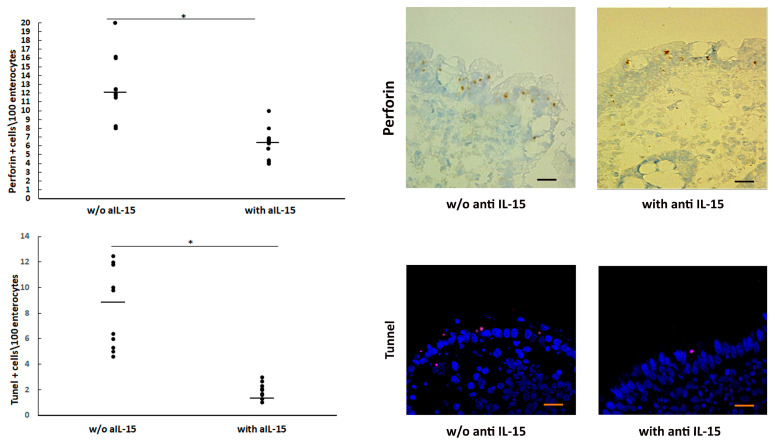
**Upper left** panel: number of perforin cytotoxic granules analyzed by immunohistochemistry, in mucosal explants from untreated CeD cultured ex vivo without (w/o) or with aIL-15 antibody. Perforin cytotoxic granules were counted in 1 mm epithelium from at least four different fields. The mean value is reported for each subject, and dashes indicate the mean values. Statistical significance was evaluated by comparing responses without or with aIL-15 antibody (* *p* < 0.01). **Upper right** panel: perforin cytotoxic granules (brown) in the epithelium of jejunal mucosa from untreated CeD patient cultured ex vivo without or with aIL-15 antibody. In the latter, a decrease in perforin cytotoxic granules (brown) in the epithelial compartment is evident. The image is representative of ten separate experiments in which biopsies taken from ten patients with untreated CeD cultured with or without the antibody aIL-15 were analyzed. Original magnification ×63; scale bar 10 µm.

One of the most potent immunological triggers for apoptosis is the release of cytotoxic granules, made up of perforin, a pore-forming protein, by activated cytotoxic CD8+ T IELs. To define whether the aIL-15 antibody might interfere ex vivo with the release of perforin in CeD intestinal mucosa, biopsies from ten untreated CeD patients were cultured in the presence or absence of an aIL-15 antibody. Perforin expression was analyzed by immunohistochemistry. As shown in Figure 1 (upper left and right panel), the number of perforin cytotoxic granules in the epithelium was significantly higher in the untreated CeD mucosa cultured without aIL-15 antibody compared to biopsies cultured with aIL-15 antibody (mean ± SD): 12.8 ± 3.8 vs. 6.1 ± 2, *p* < 0.001), while the control isotype had no effect (data not shown). To establish whether the inhibition of perforin release by aIL-15 antibody affected cell apoptosis, experiments were performed with the TUNEL assay. The density of apoptotic cells in the epithelial compartment was expressed as the number of stained cells per mm of epithelium. In biopsies challenged in vitro with the aIL-15 antibody, the number of apoptotic cells significantly decreased in comparison with biopsies cultured without the aIL-15 antibody (2 ± 0.8 vs. 8.25 ± 3.30, *p* < 0.01) (Figure 1 lower left and right panel).

### 3.2. Lower Left Panel: Overall Results of TUNEL+ Cells Counted in the Epithelium of Organ Cultured Biopsy Specimens

TUNEL+ cells were counted in 1 mm epithelium from at least four different fields. The mean value is reported for each subject, and dashes indicate the mean values. Statistical significance was evaluated by comparing responses without or with aIL-15 antibody. * *p* < 0.01. Lower right panel: TUNEL+ cells in the epithelium of jejunal mucosa from untreated CeD patient cultured ex vivo without or with the aIL-15 antibody. In the latter, a decrease in TUNEL+ cells (pink) in the epithelial compartment is evident. The image is representative of ten separate experiments in which biopsies taken from ten patients with untreated CeD cultured with or without the aIL-15 antibody were analyzed. Original magnification ×63, scale bar 10 µm.

### 3.3. Decreased FAS and HLA-E Expression in the Intestinal Mucosa of Untreated CeD Challenged with aIL-15

**Figure 2:**
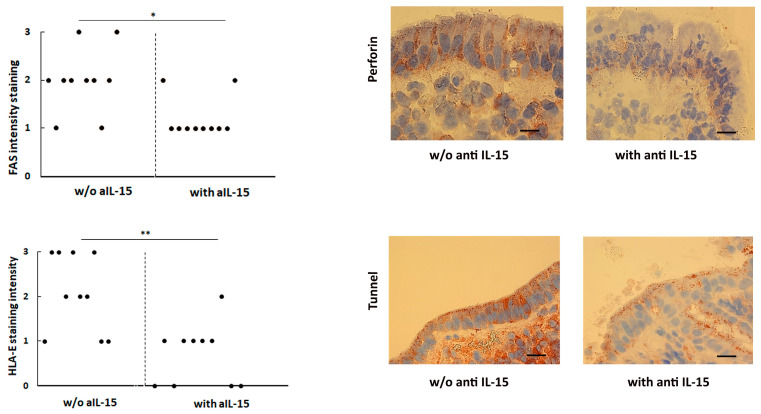
**Upper left** panel: Fas epithelial expression is decreased in the surface epithelium of duodenal mucosa of untreated CeD cultured with aIL-15 antibody. Fas expression in intestinal surface epithelium was evaluated in terms of staining intensity and graded on an arbitrary scale of staining from 1 to 3. The criteria for epithelium staining were as follows: weak staining (+) = 1, moderate staining (++) = 2, and strong staining (+++) = 3. Circles represent the response from individual patients. * *p* < 0.01, ** *p* < 0.001. **Upper right** panel: Fas expression in the epithelium of jejunal mucosa from n = 10 untreated CeD cultured ex vivo without (w/o) or with aIL-15 antibody. In the latter, lower staining is detected, particularly in almost all the epithelial cells. Original magnification ×63, scale bar 10 µm.

Several studies reported a marked increase in the cell surface expression of Fas and HLA-E in the intestinal mucosa of untreated CeD patients. Such molecules mediate the killing of enterocytes by activated CD8+ T IELs. Therefore, the effects of the aIL-15 antibody on Fas and HLA-E epithelial expression were investigated. Staining of intestinal epithelial cells expressing Fas or HLA-E was graded as weak, moderate, or strong. As expected, the enterocyte expression of both Fas and HLA-E was higher in untreated CeD patients compared to the control mucosa (see Supplementary Materials, Figure S1). Notably, the enterocyte expression of both Fas (Figure 2 upper left and right panel) and HLA-E (Figure 2 lower left and right panel) were significantly decreased in untreated CeD mucosa cultured with the aIL-15 antibody compared to the biopsy specimens cultured without aIL-15 (*p* < 0.01 and *p* < 0.001, respectively), while the control isotype had no effect (data not shown).

### 3.4. Lower Left Panel: HLA-E Epithelial Expression Is Decreased in the Surface Epithelium of Duodenal Mucosa of Untreated CeD Cultured with aIL-15 Antibody

HLA-E expression in the intestinal surface epithelium was evaluated as described for Fas in the upper left panel. ** *p* < 0.001. Lower right panel, HLA-E expression in the epithelium of jejunal mucosa from untreated CeD cultured ex vivo without or with the aIL-15 antibody. (In the latter, lower staining of HLA-E is detected, particularly in almost all of the epithelial cells.) Original magnification ×63, scale bar 10 µm. The example, for both Fas and HLA-E, is representative of n = 10 separate experiments, in which biopsies taken from patients with untreated CeD cultured with or without the aIL-15 antibody were analyzed.

### 3.5. Neutralizing IL-15 Downregulates IFN-γ Expression in Untreated CeD Patients

Cytotoxic CD8+ T IELs, once activated by several mediators, such as IL-15, in addition to releasing cytotoxic molecules, synthesize IFN-γ, which regulates the expression of several enterocyte cell-surface receptors, such as HLA-E and Fas. To study the ability of the aIL-15 antibody to prevent IFN-γ production in the epithelium of untreated CeD mucosa, duodenal biopsies from six untreated CeD patients were cultured ex vivo with or without the IL-15 neutralizing antibody.

To study the ability of the aIL-15 antibody to prevent IFN-γ production in the epithelium of untreated CeD mucosa, duodenal biopsies from untreated CeD patients were cultured ex vivo with or without the IL-15 neutralizing antibody. Such analysis was performed only on six of the ten recruited patients since, for the other patients, either the biopsy cultured without or with the aIL-15 antibody was completely used for immunohistochemistry analysis.

**Figure 3:**
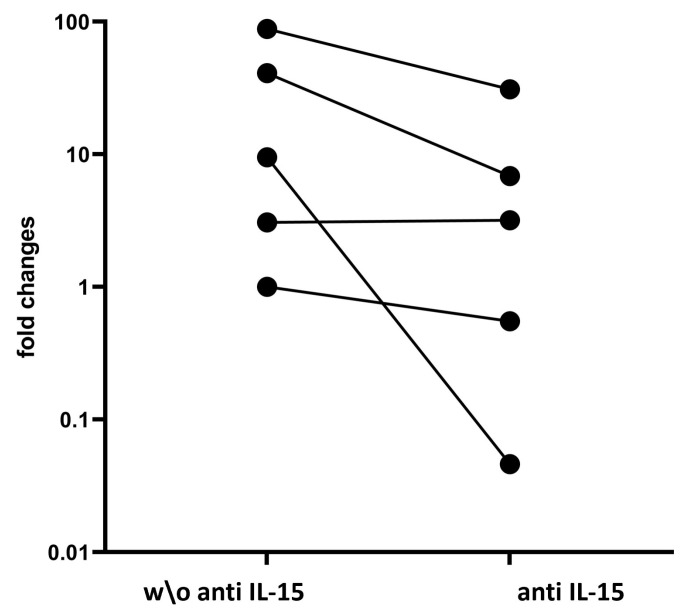
Relative levels of IFN-γ in the surface epithelium (Ep) compartment isolated by LCM from jejunal biopsies cultured ex vivo without (w/o) or with aIL-15 antibody. The biopsies were analyzed by RT-qPCR from the untreated CeD. The fold change represents the relative expression of IFN-γ mRNA normalized to GAPDH. Each point on the plot is representative of a distinct patient (*n* = 5). The lines link the IFN-γ gene expression of each subject in the two different experimental conditions.

Of note, as previously reported [18], in untreated CeD mucosa, the surface epithelium exhibits an increase in mRNA level of IFN-γ compared to that from healthy subjects. Here, a significant decrease in the transcripts levels of the pro-inflammatory cytokine IFN-γ in the surface epithelium of untreated CeD mucosa cultured with aIL-15 antibody compared to the surface epithelium isolated from jejunal biopsies cultured without aIL-15 antibody was found (*p* < 0.01) (Figure 3).

## 4. Discussion

It is well known that the massive intraepithelial infiltration of CD8+ T lymphocytes into the intestinal mucosa of CeD patients has a prominent role in the generation of intestinal villous atrophy. Activation of CD8+ IELs is most likely mediated through IL-15, an inflammatory cytokine that is highly upregulated in duodenal mucosa of untreated CeD patients [19].

**Figure 4:**
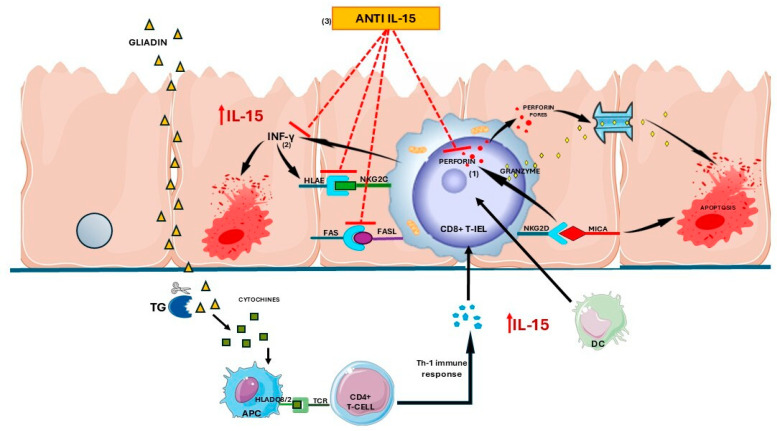
A possible model whereby anti-IL-15 may block apoptosis mediated by cytolytic mechanisms in celiac disease. (1) CD8+IELs, activated by IL-15, synthesize cytotoxic molecules such as perforin that cause the cytolysis of enterocytes. (2) Moreover, activated CD8+IELs synthesize IFN-γ, which induces enterocyte HLA-E and Fas expression. HLA-E and Fas can combine with the CD8+ IELs cell-surface receptors CD94/NKG2C and FasL, respectively, contributing to enterocyte apoptosis. (3) Anti-IL-15 may limit cytotoxic T-cell function by inhibiting the expression of perforin, HLA-E, Fas and IFN-γ.

Once triggered, CD8+ IELs produce cytotoxic molecules, such as perforin and granzyme B, and synthesize IFN-γ, which induces HLA-E expression on enterocytes. HLA-E, a non-classical MHC class I molecule, can combine with the HLA-E receptor CD94/NKG2C expressed on the surface of CD8+ IELs, contributing to enterocyte apoptosis [10,11,12] (Figure 4).

Therefore, it has been proposed that IL-15 plays a key role in immune-mediated tissue destruction [9], and treatments aimed at neutralizing its effects could be considered strategic in normalizing intestinal immune homeostasis in CeD patients.

Preclinical translational studies in humans have suggested therapeutic potential for blocking IL-15 in CeD. Lähdeaho et al. [20] investigated the safety and efficacy of AMG 714, an anti-IL-15 monoclonal antibody, also known as PRV-015, in patients with CeD undergoing a gluten challenge, showing no change in the villous height–crypt depth (Vh:Cd) ratio from baseline after 12 weeks of treatment between the placebo and treated groups. Subsequently, Cellier et al. [21] investigated the effect of the same AMG 714 in the treatment of patients with refractory CeD type 2, showing benefits in IEL T-cell receptor clonality and in the reduction of diarrhea in some patients. However, this treatment failed to prevent mucosal damage, indicating that further study of AMG 714 in refractory CeD type 2 is warranted. A phase IIb trial to determine the safety and efficacy of three dose levels of AMG 714 versus placebo in adult patients with non-responsive CeD as an adjunct to GFD has been completed and the results are currently awaited (PRV-015; ClinicalTrials.gov, Number: NCT04424927).

In our study, using intestinal mucosa cultured ex vivo from untreated CeD patients, we analyzed, for the first time, the effect of IL-15 inhibition on the epithelial expression of molecules involved in T cell-mediated cytotoxicity, such as perforin, HLA-E and Fas. In addition, cell apoptosis by TUNEL assay was performed. Furthermore, the effects of the aIL-15 antibody on the expression level of IFN-γ were studied, considering the triggering effects of this cytokine in cytotoxicity.

We observed a reduction in perforin cytotoxic granules in the epithelium of biopsy specimens challenged with aIL-15 compared with those cultured in medium alone. Perforin is a pore-forming protein found within the granules of cytolytic CD8+ T cells. Its function is to mediate the formation of transient pores on the surface of the target cells which allow the entry of granzyme B into the cytosol, leading to apoptosis of target cells [22]. Moreover, as was previously shown, we confirmed both an increased percentage of TUNEL+ apoptotic cells in the surface epithelium of jejunal biopsies of untreated CeD with respect to healthy subjects [23] and a decreased enterocyte apoptosis in biopsies cultured with the anti-IL15 antibody [3].

We also demonstrated that anti-IL-15, in intestinal mucosa from untreated CeD patients, causes downregulation of molecules that mediate the killing of enterocytes by activated CD8+ T IELs, such as Fas and HLA-E, compared with biopsy specimens cultured without the aIL-15 antibody. Fas is a cell surface receptor expressed on many cells, including enterocytes, which, after the interaction with its natural ligand FasL, mediates apoptosis [24]. The lower expression of Fas on epithelial cells from untreated mucosa challenged with aIL-15 suggested a possible reduced interaction with FasL. However, experiments are underway to prove this phenomenon.

An increased expression of both perforin and Fas in untreated CeD mucosa supports the possible involvement of these two cytolytic mechanisms in apoptosis. Importantly, in this study, we demonstrated that blocking IL-15 ex vivo in untreated CeD mucosa can prevent both cytolytic mechanisms. Furthermore, we showed that epithelial HLA-E expression was reduced in biopsy samples cultured with aIL-15. HLA-E on enterocytes can combine with the HLA-E receptor, NKG2C, expressed on the surface of CD8+ IELs, contributing to the cytolysis of enterocytes [10,11,12]. Additionally, it was observed that the enterocyte expression of HLA-E is induced by IFN-γ produced by activated CD8+ IELs.

As previously reported [18], the epithelial compartment, isolated by LCM, of jejunal biopsies of untreated CeD showed an increased expression of IFN-γ with respect to healthy subjects. To determine if IL-15 inhibition impacts IFN-γ and HLA-E expression, we examined IFN-γ expression in the epithelium of untreated CeD biopsies, both with and without aIL-15 antibody treatment. A significant reduction in IFN-γ expression was observed in the epithelial compartment of biopsy specimens challenged with the aIL-15 antibody relative to those cultured without the aIL-15 antibody. These data could explain the decreased expression of HLA-E on epithelial cells from jejunal biopsies of untreated CeD challenged ex vivo with aIL-15.

In addition to the crucial role in activating the cytotoxic CD8 and NK cells leading to villous atrophy, we and others have shown that IL-15 also mediates the activity of anti-inflammatory pathways, including those mediated by T reg cells [13,14], thus contributing to the loss of intestinal homeostasis and promoting chronic inflammation in CeD. Benahmed et al. [13] have shown that, in ex vivo cultured untreated celiac intestinal mucosa, IL-15 is involved in the local downregulation of TGF-β signaling and that anti-IL-15 antibodies restore TGF-β effects. Therefore, the inhibition of such a cytokine may not only suppress cytolytic mechanisms but also promote immune tolerance at the inflamed tissue by promoting the restoration of regulatory T-cell function.

The study results should be interpreted with several limitations. Firstly, the small number of biopsy samples, due to ethical issues, did not allow us to set up further experimental points to better describe the mechanism involved in IL-15 neutralization. Secondly, the ex vivo organ culture, which cannot exceed 20–24 h, allowed us to investigate only an early mucosal immune response. Additionally, given the role of overactive IL-15 signaling in CeD inflammation, an intriguing avenue for future research would be to assess the impact of IL-15 inhibition on the levels of downstream inflammatory cytokines.

## 5. Conclusions

IL-15 can participate in multiple steps of CeD pathogenesis. It was shown that the upregulation of IL-15 in the gut epithelium might drive the accumulation of IEL in untreated CeD and in RCDII [25,26]. Furthermore, IL-15 can induce CD8+ T IELs to kill epithelial cells based on stress signals, which would result in increased intestinal permeability through tight junction protein imbalances. Several pieces of data indicate that the cooperation between cytokines released by gluten-specific CD4+ T cells (i.e., IFN-γ, IL-21, IL-2) and IL-15 released by dendritic cells is also necessary to activate CD8+ T IELs, which drive tissue damage in untreated CeD [27]. We and others [13,14] have provided evidence that IL-15 can impair local immunoregulation, impeding the Smad3 pathway of TFGβ and interfering with regulatory T cells’ suppressive capacity. Together, these results provide a rationale for a strategy consisting of blocking IL-15 signals to reduce both IEL accumulation and their cytolytic capacity as well as to restore immunoregulation.

In summary, our findings suggest a scenario whereby anti-IL15 may prompt a block of apoptosis both by reducing the production of cytotoxic granules and the Fas–FasL/HLA-E-CD94/NKG2C cognate interaction (Figure 4). These results underscore the potential of IL-15-targeted therapies at preventing or repairing intestinal damage induced by gluten and thus represent a promising strategy for the treatment of CeD or inflammatory diseases associated with the overexpression of IL-15.

Further investigation into the precise mechanisms underlying IL-15-mediated cytotoxicity as well as on the restoration of the TGF-β regulatory function as well as the development of specific IL-15 inhibitors could lead to novel therapeutic approaches for this disease. 

## Figures and Tables

**Table 1:** qRT-PCR primers used in this study.

Gene	Accession Number	Oligonucleotide Sequences (5′ → 3′) Forward Primer	Oligonucleotide Sequences (5′ → 3′) Reverse Primer
IFN-γ	NM_000619	GTTTTGGGTTCTCTTGGCTGTTA	AAAAGAGTTCCATTATCCGCTACATC
GAPDH	NM_002046.2	ATGACATCAAGAAGGTGGTG	CATACCAGGAAATGAGCTTG

## Data Availability

The data presented in this study are available from the corresponding author upon request.

## Supplementary Materials

The following supporting information can be downloaded at: https://www.mdpi.com/article/10.3390/cells14030234/s1, Figure S1: Immunohistochemical staining for Fas and HLA-E from untreated CeD and healthy subjects intestinal tissue sections. Table S1: Phenotypic data of untreated CeD patients.
