# Supplementary material for: Neutralizing IL-15 Inhibits Tissue-Damaging Immune Response in Ex Vivo Cultured Untreated Celiac Intestinal Mucosa
Source: Cells. 2025 Feb 6;14(3):234. doi: 10.3390/cells14030234 (PMC11818035; doi:10.3390/cells14030234)
Supplement: Supplementary file 1 [file cells-14-00234-s001.zip › cells-3403203-supplementary.pdf]

Supplemental material

Table S 1. Phenotypic data of untreated CeD patients.

Patients	Gender	Age	EMA antibodies	Histology
#1	F	44	Positive	Marsh II
#2	F	39	Positive	Marsh III b
#3	M	18	Positive	Marsh IIIc
#4	F	43	Positive	Marsh IIIc
#5	F	45	Positive	Marsh II-III
#6	F	39	Positive	Marsh IIIc
#7	M	41	Positive	Marsh III b
#8	F	42	Positive	Marsh IIIc
#9	F	34	Positive	Marsh IIIc
#10	M	45	Positive	Marsh IIIc

Figure S 1.

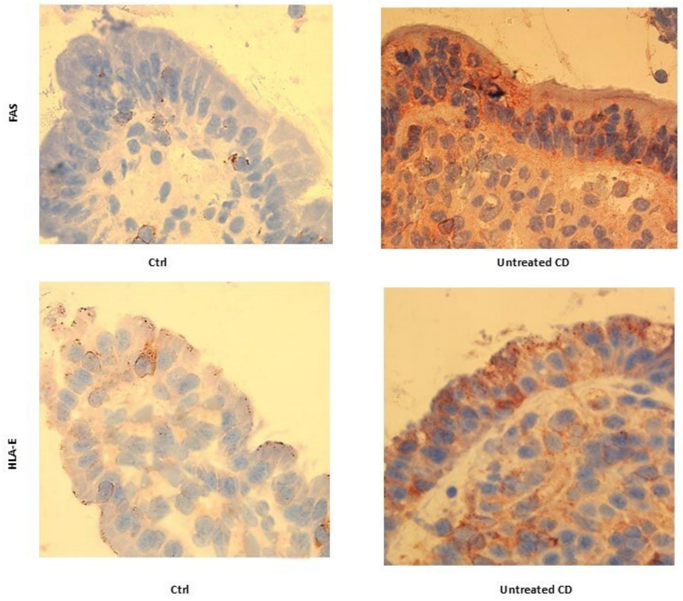

Immunohistochemical staining for Fas (upper panel) and HLA-E (lower panel) from untreated CeD and healthy subjects intestinal tissue sections. Both Fas and HLA-E staining is less diffuse and intense than that observed in untreated CeD. The example is representative of ten separate experiments, in which biopsies taken from ten patients with untreated CeD and ten healthy subjects were analyzed. Original magnifications, x63.
